# Thermal Conductivity of Polyvinylidene Fluoride Films with a Multi-Scale Framework

**DOI:** 10.3390/polym15102331

**Published:** 2023-05-16

**Authors:** Qin Wang, Shixin Liu, Hong Guo, Boyang Hu, Yi Li, Jixiao Wang, Baoan Li

**Affiliations:** 1 Chemical Engineering Research Center, School of Chemical Engineering and Technology, Tianjin University, Tianjin 300350, China; 2 State Key Laboratory of Chemical Engineering, Tianjin University, Tianjin 300350, China; 3 Tianjin Collaborative Innovation Center for Chemistry & Chemical Engineering, Tianjin 300350, China; 4 Department of Energy and Chemical Engineering, Tianjin Ren’ai College, Tianjin 301636, China; 5 College of Environmental Science and Engineering, Nankai University, Tianjin 300350, China

**Keywords:** thermal conductivity, structural manipulation, anisotropic amorphous, multi-scale framework

## Abstract

The orientation of amorphous regions in pure polymers has been noted to be critical to the enhancement of thermal conductivity (TC), but the available reports are still rather few. Here, we propose to prepare a polyvinylidene fluoride (PVDF) film with a multi-scale framework by introducing anisotropic amorphous nanophases in the form of cross-planar alignments among the in-planar oriented extended-chain crystals (ECCs) lamellae, which show an enhanced TC of 1.99 $Wm^{-1}\;K^{-1}$ in the through-plane direction ($K_{⊥}$) and 4.35 $Wm^{-1}\;K^{-1}$ in the in-plane direction ($K_{∥}$). Structural characterization determination using scanning electron microscopy and high-resolution synchrotron X-ray scattering showed that shrinking the dimension of the amorphous nanophases can effectively reduce entanglement and lead to alignments formation. Moreover, the thermal anisotropy of the amorphous region is quantitatively discussed with the aid of the two-phase model. Superior thermal dissipation performances are intuitively displayed by means of finite element numerical analysis and heat exchanger applications. Moreover, such unique multi-scale architecture also results in significant benefit in the improvement of dimensional stability and thermal stability. This paper provides a reasonable solution for fabricating inexpensive thermal conducting polymer films from the perspective of practical applications.

## 1. Introduction

With the miniaturization and integration of electronic devices, a serious issue concerning heat dissipation has been gradually triggered, resulting in reduced reliability and shortened service life of devices. In addition, heat exchangers for industrial, heating, air conditioning (HVAC), and desalination applications seek better chemical and pollution-resistant materials and emphasize greater design freedom [1]. Traditional metal heat-dissipating materials (HDMs) fail to satisfy the modern requirements of easy corrosion, high density, and difficult processing. Novel HDMs possessing high thermal conductivity (TC), corrosion resistance, low cost, and low density are facing unprecedented demands and development opportunities [2]. Polymer materials have been widely applied in modern technologies due to their unique advantages [3,4,5,6]. However, the very low TC (~0.1 $Wm^{-1}\;K^{-1}$) [7] largely hampered their wide development in the thermal management fields. The most popular solution was to modify polymeric matrices into thermally conductive composites [8,9]. With the addition of high TC fillers, such as carbon-based materials [10,11], metallic particles [12,13], and ceramic particles [14,15], percolating thermal conductive networks can be formed to enhance TC. Afterwards, multiple orientation approaches, such as field orientation [16,17] and the template method [18,19], were utilized to optimize thermal conductive fillers into specific oriented thermal conductive channels. In addition, many other hybrid methods can also be utilized to obtain composites of thermally conductive polymers [20,21]. Polymer-based composites can exhibit improved TC. Nonetheless, either an expensive additive or a complex fabrication process is indispensable to bring about realistic issues of cost increasing and environmental pollution.

Unlike the entangled morphology, single-chain polymers composed of carbon-carbon covalent bonds have been reported to have inherently high TC in the direction of linear orientation [22,23]. Some advanced technologies, such as ultra-stretching [24,25], nano-templating [26], and electro-spinning methods [27], have also been reported for preparing ultra-high TC nanostructures in pure polymers successfully based on the premise of realizing ultra orientation. These nanostructures derived from the chain-oriented morphology of the molecular assemblies are in the forms of nanocrystalline fibers, lamellar crystals, and nano alignments of amorphous chains [28]. Intuitively, it could be understood that the foundation of preparation strategies for pure polymers is to minimize chains entanglements in the view of structural manipulation to achieve high TC. Notably, crystal structures in the form of oriented extended-chain crystals (ECCs) have fewer numbers of entanglements and can be used to enhance TC by simply increasing the crystallinity degree. In addition, the longer folded lengths of the lattice chains in ECCs can also further reduce the entanglements [29,30]. More strikingly, metallic-like TC of linear UHMWPE taps and film can be obtained by simultaneously aligning the crystal chains and amorphous chains in a single direction [31]. Afterwards, such remarkably high TC has been elucidated owing to the ultra-orientation of amorphous chains [23]. It is clear that achieving anisotropy or even uniform orientation in the amorphous regions is crucial for the preparation of pure polymer thermal conductive materials.

However, most of the studies aimed to improve TC in pure polymers have two major limitations that need to be addressed. First, the previous series of reports of the TC enhancement of pure polymers were mainly limited to PE with different molecular weights. The study of the oriented amorphous morphology of polymers other than PE is also meaningful for the development of pure polymers with improved TC. Second, the structural manipulation in the reported literature always focused on in-plane orientation. Correspondingly, TC in through-plane contrarily tends to decrease or remain unchanged during the structure formation. This feature can be explained in terms of the reduction of intra-chain covalent bonding in the through-plane direction. Simultaneously improving in-plane and through-plane TC on the basis of multi-scale structural modulation is of vital important to expand the range of applications for practical thermal management, and relevant research has been carried out in the aspect of thermal conductive composites [32]. Nevertheless, the preparation and discussion of multiscale frameworks in pure polymers has not been reported yet, which is preferable in the form of an oriented structure in the through-plane while ensuring the in-plane orientation structure.

Polyvinylidene fluoride (PVDF) is a thermoplastic polymer that has long been widely used in various fields such as the chemical, power and electronics, water treatment, and food industries [33]. In addition, the excellent properties of chemical and thermal stability as well as good mechanical and weather resistance of PVDF have also aroused a lot of interests in the field of thermal conductive composites [15,34], for which it is an attractive material in the scope of the development of next-generation electronics and heat exchanger applications. As a semi crystalline polymer, the presence of nanostructured crystallites embedded in the PVDF matrix make it possible for the persistent orientation of amorphous molecules induced by orientation of the crystallites. Therefore, we proposed a dual-assembly strategy to fabricate PVDF film with a multiscale framework, which not only has ECCs lamellae oriented in in-plane, but more importantly, the amorphous regions are vertically aligned in the through-plane direction in a form of anisotropic nanophase structures. Rather than focusing on reducing entanglement from the perspective of individual polymer chains, this unique architecture is inspired by the high TC of polymer nanostructures, which consist of oriented chain assemblies with less entanglement and easy to orient. As a result, the TC was finally improved to 1.99 $Wm^{-1}\;K^{-1}$ in the through-plane direction and 4.35 $Wm^{-1}\;K^{-1}$ in the in-plane along the stretching direction. More than that, we conducted a qualitative discussion using the measured TC values to further understand the thermal anisotropy of the amorphous region on the basis of the two-phase constituents. Intuitive finite element numerical analysis and practical heat exchanger application experiments were adopted to reveal the structure advantage in practical thermal applications.

## 2. Materials and Methods

### 2.1. Materials

Commercial PVDF (Grad FR906) particles were purchased from 3F New Material Co., Ltd., Shanghai, China. Dimethyl formamide (DMF) was purchased from Jiangtian Technology Co., Ltd., Tianjin, China. All the materials were used without purification.

### 2.2. Polymer Processing

**Figure 1:**
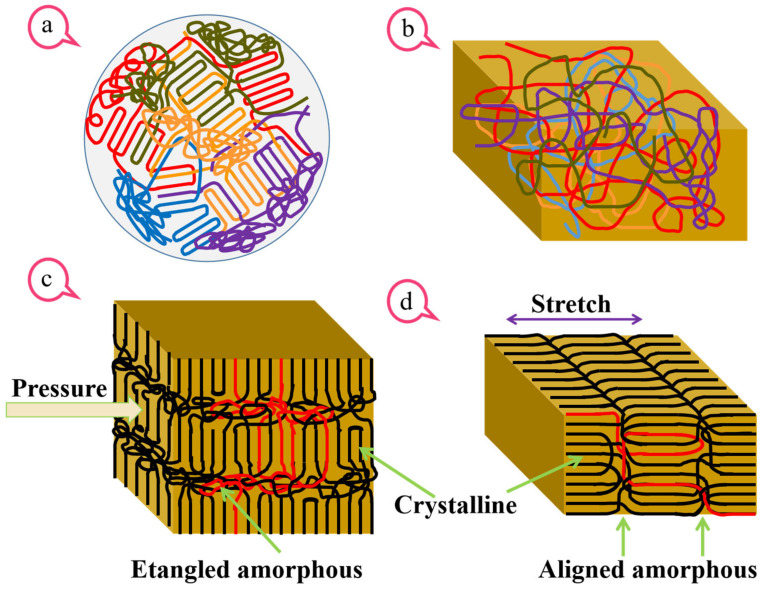
Brief illustrations of the thermal conduction pathway (red curve) correspond to every molecular chain transformation. (**a**) Polyvinylidene fluoride molecular chains in the particles featured with lamellar crystallites embedded in a disordered and entangled chain network; (**b**) The molecular chains were fully interlaced in the hot DMF solution; (**c**) The polymer chain segments were assembled into extended-chain crystallites as vertically arranged lattice chains under a horizontal pressurization; (**d**) The BONs film is characterized by parallelly oriented crystallites interconnected by vertically aligned amorphous chains.

For pure polymers, the multiscale framework is essentially a network structure composed of molecular chains and requires the existence of intermolecular interaction in multiple directions. To ensure this, the manufacturing process of a dual-assembly strategy will be divided into two parts. Figure 1 schematically presents the corresponding heat conduction path of each molecular chain transition.

In the initial part, the polymer chains in the applied template need to have a relatively strong intermolecular interaction effect between polymer chains so that the network structure can be formed uniformly. PVDF particles, incorporating a chaotic structure of amorphous domains embedded with lamellar crystallites (Figure 1a), were dissolved in DMF solvent at 60 °C for 2 h at a 20% weight ratio condition, then cast into square substrate molds and cooled in a liquid nitrogen environment directly. After evaporating the solvent under vacuum for 72 h, the cast parison featured with a fully interlaced network (Figure 1b) was obtained.

In the second part, the as-prepared parison was sealed into the horizontal slit of a home-made piston-slit device to melt at 0.1 MPa and 220 °C for 20 min. After that, the temperature was reduced to 150 °C and subjected a horizontal pressure along the slit entrance for 60 min, by which the polymer chain segments can be assembled into extended-chain crystallites with vertically arranged lattice chains (Figure 1c) [34,35]. Then, the melt was extruded into the air (25 ± 3 °C) and stretched horizontally to a uniform state to cold. Melt deformation allowed not only rearranging the lamellar lattices into in-plane assembled ECCs lamellae but also tauting and disentangling the amorphous tie of molecules between different lattice layers, and finally forming an assembly nanophase structure of amorphous alignment in the through-plane direction (Figure 1d). Three pressure conditions (10, 50, and 100 MPa) were adopted to track the evolution of those two bi-planar oriented nanostructures (BONs) in the multi-scale framework, and the corresponding samples were named as BONs-1, BONs-2, and BONs-3 for brevity. The constant temperature and pressure conditions were controlled using two proportional-integral-derivative (PID) controllers with respective inaccuracies of ±0.1 °C and ±0.035 MPa.

## 3. Characterizations

The surface and cross-section morphology were observed with Scanning Electron Microscope (SEM) Observation (S-4800, Hitachi Ltd., Tokyo, Japan). The tested cross-sectional samples were quenched in liquid nitrogen. All samples were observed after 40 s of gold spraying process (B7340/109, Agar scientific, Essex, UK). The orientation of the molecular chain was tested using high-resolution wide-angle and small-angle synchrotron X-ray scattering (WAXS and SAXS) from the Chinese Academy of Sciences. The long period (L) was calculated using the Bragg equation of L=2π/q, where q is the peak position in the 1D SAXS scattering curves. TC (κ) at room temperature (25–30 °C) was calculated from the equation: κ = $\rho \alpha c_{p}$. Density (ρ) was measured using a densimeter (XS205, Mettler Toledo, Greifensee, Switzerland) with ethanol as the reference (ASTM D792). Thermal diffusivity (α) was evaluated via the laser flash method through the NETZSCH LFA467 hyperflash instrument (NETZSCH, Selb, Germany), and each sample was carbon coated before the measurement (Black Guard Spray FC153, Fine Chemical Japan Co., Ltd., Tokyo, Japan). (See electronic supplementary materials for an overview of TC determination methods.) Thermal expansion rates were determined using the NETZSCH TMA 402F3 instrument with a heating rate of 5 °C min^−1^ in a temperature range of 20 to 100 °C. Differential scanning calorimeter of NETZSCH DSC 214 was performed with a heating rate of 10 °C min^−1^ in the temperature ranged from room temperature to 200 °C in a nitrogen atmosphere. The thermal stability was investigated using a DTG-60AH differential thermal thermogravimetric synchronization analyzer (Shimadzu, Kyoto, Japan) with a heating rate of 10 °C min^−1^ in the temperature ranged from room temperature to 750 °C in an inert atmosphere.

## 4. Results and Discussion of BONs

### 4.1. SEM Morphology of BONs Films

Distinguished variation in the surface and cross-section morphology from BONs-1 to BONs-3 revealed the formation of multi-scale framework under polymer chain-base assemblies.

**Figure 2:**
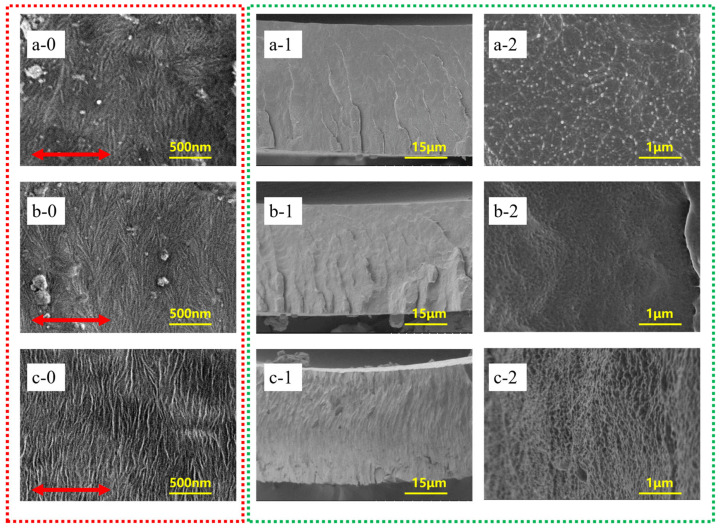
SEM pictures of the PVDF films. Note: **a**–**c** represent BONs-1~3 films, red arrows indicate the stretching direction, **0**–**2** indicates the surface morphology and two different magnifications in the cross-section.

In the observation of sample BONs-1, the ECCs lamellae began to appear on the surface morphology (Figure 2(a0)), but only a small amount of vertical texture appeared on the cross section (Figure 2(a1)). In the high-magnification image (Figure 2(a2)), some folded chain ends of crystal lattices are dispersed in the entangled amorphous matrix to separate the domain region into nanophase structures. No orientation can be seen. By comparison, largely increased ECCs lamellae with a much more clear morphology were shown on the surface of sample BONs-2 (Figure 2(b0)). In addition, there are significantly more vertical textures in the cross section of the low-magnification image (Figure 2(b1)), and more folded chain ends can be seen in the high-magnification image (Figure 2(b2)), which further separate the amorphous domain into smaller and less entangled nanophase structures. It can be seen that this double-assembly strategy can effectively reduce the entanglements by increasing the amount of in-planar oriented ECCs lamellae. On the other hand, the size reduction of amorphous nanophase structures can also contribute effectively to the entanglement reduction [36]. When observing the sample BONs-3, not merely was the surface particularly arranged with parallelly orientated ECCs lamellae (Figure 2(c0)), but also the cross-section exhibits a clear-cut of long-range ordered morphology (Figure 2(c1)), especially no chaotic entanglement was observed in the high-magnification image (Figure 2(c2)), that is, the cross-planar nanophase structure of amorphous alignment is formed. Hence, the multi-scale framework compose of BONs was successfully prepared.

### 4.2. Atomic-Scale and Nanoscale Structure of BONs Films

**Figure 3:**
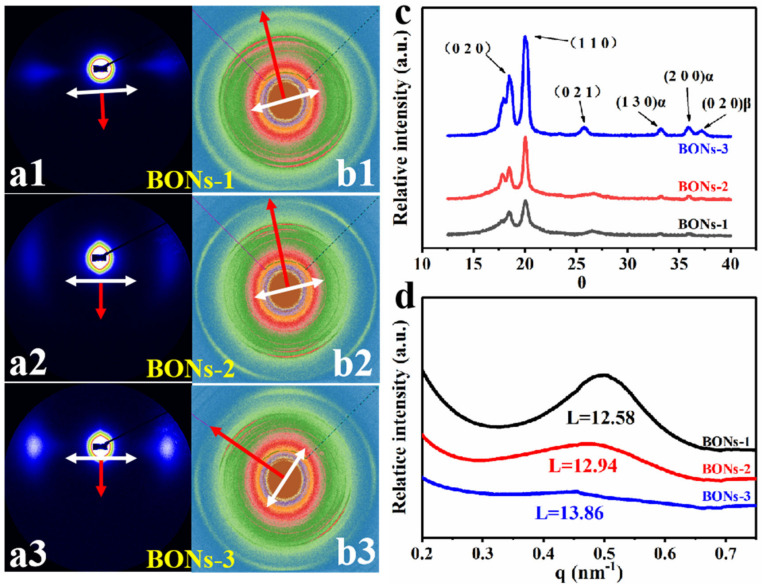
The results of high-resolution synchrotron X-ray scattering. (**a1**–**a3**) The 2D patterns of SAXS, (**b1**–**b3**) 2D patterns of WAXS, in which the white arrow indicated the stretching direction and the red arrow indicated the meridian direction. (**c**) The 1D intensity curves obtained from circular integration of 2D-WAXS scattering. (**d**) The 1D intensity profiles obtained from circular integration of 2D-SAXS patterns.

The above observations convinced us that the BONs film is composed of parallel ECCs lamellae in the in-plane direction and amorphous alignments in the through-plane direction. To further confirm the structure variations and the chain orientations of ECCs lamellae in the multi-scale framework, we have carried out both atomic scale and nanoscale investigations through high-resolution synchrotron X-ray scattering. All the 2D pattern results (Figure 3a,b) were detected from the in-plane direction of each sample. The white arrows and red arrows represent the meridian direction and stretching direction, respectively.

The variation of the symmetric signal reflections in the 2D-SAXS patterns (Figure 3(a1–a3)) implied a formation of gradually parallelled oriented morphology of lamellar ECCs in the in-plane direction [37]. After circular integration of these 2D SAXS patterns, the obtained 1D intensity profiles (Figure 3d) appeared with a hump in each curve, which demonstrated the repeat unit of the present ECCs lamellae. Moreover, the weakened intensity of the humps suggested a gradual uniform lamellae structure in the preparation process [30,38]. In addition, the peak of the curve from sample BONs-1 to BONs-3 has a slight leftward shift tendency, which corresponded to the insignificant increasing change of period length from 12.58 nm to 13.56 nm. It follows the fact that as the crystallinity increases, more ECCs lamellae will be formed, as well as more crystal-amorphous interphases for the dispersing of the reduced amorphous region. On basis of that, the size of the amorphous nanophase structures between the lamellar ECCs must be reduced, which is in good agreement with the morphological variations observed using SEM.

The 2D Wide angle X-ray scattering (WAXS) patterns of samples BONs-1, BONs-2, and BONs-3 (Figure 3(b1–b3)) exhibit the gradually clear and refined scattering arcs of the {hk0} groups. It illustrates the improvement in the crystallization and the increased degree of crystallinity for one thing. Meanwhile, it is worth noting that the one-dimensional WAXS intensity profiles (Figure 3c) show distinct 2θ peaks in the angular range of 18.0°–20.1°, corresponding to the (020) α and (110) α reflections [39,40,41]. Specifically, the arcs of the {hk0} groups of the three samples present a symmetrical contraction along the meridian direction, indicating that the orientation of the c-axis (lamellar lattice) along the stretching direction is improved [23,34], although some other negligible characteristic reflections with relatively weak intensity can also be detected, such as the (200) β reflection [42]. Therefore, the multiscale framework is identified to be a polymer chain-based structure consisting of an in-plane oriented crystal axis and through-plane-arranged amorphous chains.

### 4.3. Thermal Anisotropy Analysis of the Amorphous Region

Based on the existence of through-plane aligned amorphous nanophase structures among the in-plane ECCs lamellae in the characteristic multiscale framework, the BONs film is expected to be a promising candidate as a thermally conductive polymer with improved TC. To further understand the thermal anisotropy of the amorphous region, we used the measured TC values for qualitatively discussing the application of the two-phase model.

**Figure 4:**
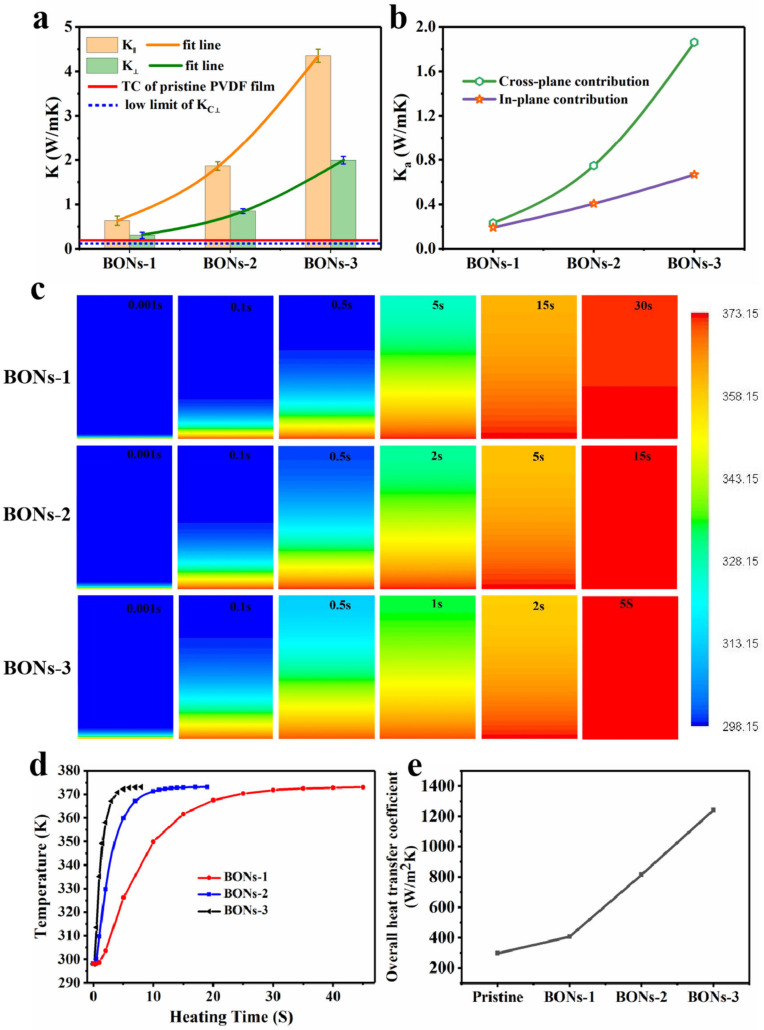
Illustration of TC analysis and heat transfer performances of BONs films. (**a**) The measured values of ${Κ}_{⊥}$ and ${Κ}_{∥}$ of BONs films. (**b**) The anisotropic ${Κ}_{a}$ values calculated using the two-phase model theory. (**c**) The one-dimensional transient thermal response of in-plane TC. (**d**) The calculated transient temperature distribution on the top side of the two-dimensional model. (**e**) The experimental heat transfer coefficients of the cross-plane TC application.

The TC values measured along the in-plane stretching direction (${Κ}_{∥}$) of samples BONs-1, BONs-2, and BONs-3 are 0.64, 1.86, and 4.35 $Wm^{-1}\;K^{-1}$, respectively (Figure 4a). As compared to the pristine counterpart (0.18 $Wm^{-1}\;K^{-1}$) [43], these higher values give an increase of 255%, 933%, and 2317%, respectively. This typical increasing behavior is due to the increase in crystallinities and orientation, by which the molecular chains of lamellar lattices are essentially parallelly oriented along the stretching direction, thus offering very little thermal resistance. However, even this gradual increase in the slope in this case is superior to that for the reported bulk materials; it is far less than the metal-like improvement obtained vi ultra orientation. It can be explained that the suppression of the increasing behavior was caused by the large thermal resistance of the scattering effects on account of the increasing boundaries between the crystalline and amorphous phases. On the other hand, the in-plane amorphous intrachain covalent bonding in the BONs framework is significantly reduced because of the cross-plane distribution of amorphous alignment. Since ${Κ}_{∥}$ is strongly dependent on the in-plane orientation of ECCs lamellae, the value of ${Κ}_{∥}$ can be evaluated using the Modified Maxwell formula as shown below [44]:

(1) $$\frac{{Κ}_{∥}-{Κ}_{a}}{{Κ}_{∥}+{2Κ}_{a}}=Χ\left[\frac{k_{⊥}-1}{k_{⊥}+2}\times \left\langle {sin}^{2}\theta \right\rangle +\frac{k_{∥}-1}{k_{∥}+2}\times \left\langle {cos}^{2}\theta \right\rangle \right]$$

where $k_{⊥}={Κ}_{c⊥}/{Κ}_{a}$, ${Κ}_{c⊥}$ is the TC perpendicular to the chain direction of crystallites, and the value adopted here was from [45]. The ${Κ}_{a}$ here is the contribution of the amorphous region in in-plane TC. Χ is the degree of crystallinity calculated from the ratio of the area and the total area related to the crystal area scattering. $k_{∥}={Κ}_{c∥}/{Κ}_{a}$, ${Κ}_{c∥}$ is TC along the chain direction of a crystallite. θ is the angle between the stretching direction and the chain axis of crystallites, and the value of ${Κ}_{c∥}$ is considered to be saturated. The values of $\left\langle {sin}^{2}\theta \right\rangle$ and $\left\langle {cos}^{2}\theta \right\rangle$ can be calculated from the crystalline orientation function of $f_{c}$ given by:

(2) $$f_{c}=\frac{1}{2}\left[3\left\langle {cos}^{2}\theta \right\rangle -1\right],\;\left(0\leq f_{c}\leq 1\right)$$

According to the Picken’s method, the degree of orientation was determined via the azimuthal integration of the $\left\{hk0\right\}$ groups [46]. Considering the existence of the major specific peaks in the one-dimensional intensity profile were almost all of the ($\left\{hk0\right\}$) groups (Figure 3c), a perfect orientation of lamellar lattices ($\left\langle {sin}^{2}\theta \right\rangle$ = 0) can be assumed for simplification. Thus, the simplest relationship of ${Κ}_{∥}$ dependency ${Κ}_{a}$ can be expressed as:

(3) $$\frac{{Κ}_{∥}-{Κ}_{a}}{{Κ}_{∥}+{2Κ}_{a}}=Χ$$

The calculated TC values of amorphous contribution in in-plane of samples BONs-1 to BONs-3 are 0.19 $Wm^{-1}K^{-1}$, 0.40 $Wm^{-1}K^{-1}$ and 0.67 $Wm^{-1}K^{-1}$ (Figure 4b), respectively.

In the through-plane direction, the measured values of improved TC ($K_{⊥}$) are 0.31, 0.85, and 1.99 $Wm^{-1}K^{-1},$ respectively (Figure 4a). According to the structural characterization results described above, such improvement is mainly attributed to the size reduction of nanophase structures in the amorphous region until the formation of alignment morphology, thus achieving the maximum increment. It is because of the one-dimensional (1D) alignment in the sense that single carbon chains only align in one dimension in the aligned direction, which can significantly limit the number of phonon scattering events with size reduction, and subsequently results in longer phonon-mean free paths and higher TC [33]. It follows the fact that the thermal conduction pathway for $K_{⊥}$ is alternately composed of lamellar lattices and amorphous alignments despite different orientations. Therefore, an anisotropic model without consideration of orientation can be used to estimate the through-plane TC of the amorphous contribution and expressed as follows [47]:

(4) $$\frac{K_{⊥}-{Κ}_{a}}{K_{⊥}+2{Κ}_{a}}=Χ\left[\frac{2}{3}\frac{k_{⊥}-1}{k_{⊥}+2}+\frac{1}{3}\right]$$

Substitute $k_{⊥}={Κ}_{c⊥}/{Κ}_{a}$ into the formula and simplify it as the following polynomial:

(5) $$2{K_{a}}^{2}+\left(K_{c⊥}+2XK_{c⊥}-2K_{⊥}\right)K_{a}-\left(1-X\right)K_{c⊥}K_{⊥}=0$$

The yielded positive root is the estimate TC contribution of ${Κ}_{a}$ in the through-plane direction, and the corresponding values of samples BONs-1 to BONs-3 are 0.23 $Wm^{-1}\;K^{-1}$, 0.75 $Wm^{-1}\;K^{-1}$ and 1.86 $Wm^{-1}\;K^{-1}$ (Figure 4b), respectively. It is noticed that the calculated ${Κ}_{a}$ in the in-plane direction increases in an almost linear trend, whereas an approximately exponential increase is observed in the cross-plane direction. The two increases are certainly demonstrating that the size reduction of amorphous nanophase structures have an anisotropic-enhancing effect on both the in-plane and through-plane TCs.

Accordingly, the one-dimensional transient thermal response of the in-plane TC (${Κ}_{∥}$) is simulated via a two-dimensional model with a size of 2 × 4 mm using the finite element analysis software (ANSYS). The calculated transient temperature distribution results of the three samples are obviously compared (Figure 4c), in which the initial temperature was set as 20 °C, and the one-dimensional thermal conduction was started from a constant bottom heating temperature of 100 °C. On the top of these temperature graphs are markings with the corresponding time points. As a result, sample BONs-3 demonstrates the fastest thermal response to reach a top-side temperature of 372.20 °C in just 5 s. However, it takes 15 s for sample BONs-2 and 30 s for sample BONs-1 to be heated to 372.88 °C and 371.77 °C, respectively. The simulation results directly reflect the difference of the in-plane thermal response speed, indicating that the increment in the in-plane oriented ECCs lamellae plays a crucial role in enhancing the TC of ${Κ}_{∥}$.

In order to demonstrate the superior through-plane TC for a practical heat exchanger application, we fabricated a homemade plate heat exchanger with an effective heat exchange area of 40 × 200 mm to carry out a water–water heat exchange experiment. In each test run, the corresponding experimental data were obtained after the cold and hot fluids, with the temperatures of 15 and 70 °C, respectively, were kept running for 2 h. It can be seen from the overall heat transfer coefficient of the heat exchange experiment that, compared with the pristine counterpart displays a value close to 300 $W/\left(m^{2}\;K\right)$, the heat transfer performance of the heat exchangers made using BONs films has significantly improved. The BON-3 film heat exchanger even achieves a heat transfer coefficient up to 1241 $W/\left(m^{2}\;K\right)$, much higher than 814 $W/\left(m^{2}\;K\right)$ of the BONs-2 film and 406 $W/\left(m^{2}\;K\right)$ of the BONs-1 film. As a result, the largest heat transfer performance of the BONs-3 film is well above that of other samples, which immediately demonstrates the crucial role of aligned amorphous nanophase structures in achieving a faster thermal transition speed, that is a high through-plane TC. (Details information of the thermal management experiments can be seen in the electronic Supplementary Materials).

### 4.4. Physical Properties of BONs Films

**Figure 5:**
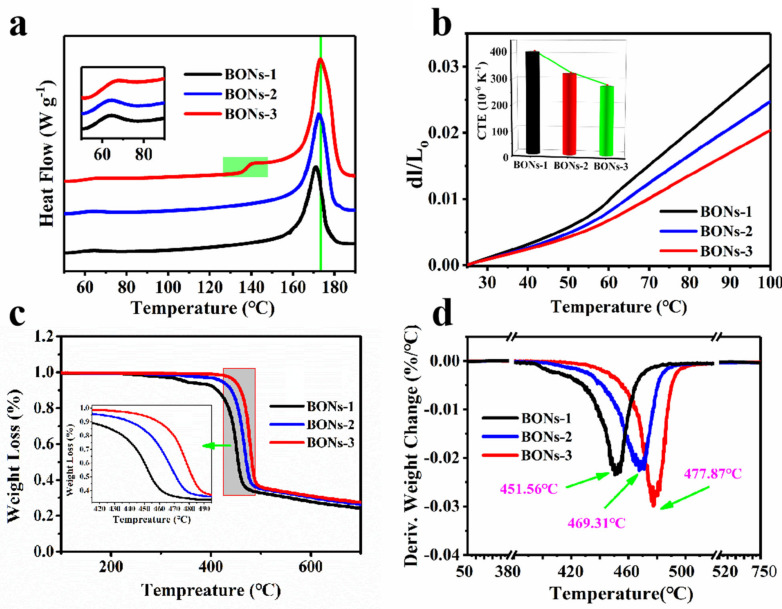
Thermostability of BONs films. (**a**) DSC thermograms, (**b**) evaluation of the thermal expansion rate. The thumbnail shows the calculated mean CTE values over the testing temperature range of 25–100 °C. (**c**) TG and (**d**) DTG thermograms.

The thermostability of the prepared BONs samples is intimately related to the existence and optimization of the multiscale framework. The DSC thermograms (Figure 5a) display a high-temperature shift of the endothermic melting peak and an increase in the endothermic heat (proportional to the peak area) from sample BONs-1 to BONs-3, indicating an increasing thermal stability below the melting region, showing the influence of the thermal-dependent melting behavior of the amorphous phase on the enthalpic relaxation process. It is recognized that the molecular motion related to the relaxation process in the amorphous region is restricted according to their proximity to the crystallites [48,49]. Consequently, far above the glass transition temperature (about −50 °C), the presence of a relatively small endothermal heat-flow peak of each specimen around the temperature of 60 °C (clearly shown in the insertion diagram) is considered as an upper glass transition owing to the $\alpha_{c}$ transition relaxation [36]. This phenomenon has always appeared in many other high-performance semicrystalline polymers as well [50,51]. Regarding the step-like transitions site of sample BONs-3 at about 130 °C, it can be explained by the growth of more tightly adjacent re-entry foldings in the geometrically restricted regions [52], which take into account secondary crystals corresponding to the relatively weak intensity of (200) β reflection.

Similar to the much more stable intra-lamellar stiffness of the crystalline phase than the amorphous phase, the slope with discontinuous changes divides each thermal expansion curve into two different growth trends (Figure 5b). Additionally, thermal expansion curves of sample BONs-1, BONs-2, and BONs-3 exhibit a successively decreasing distribution, which is due to the low to high crystallinity degrees [53]. The measurements of thermal expansions show increasing transitions that can be satisfactorily addressed using a mean CTE determined for a temperature range of 25–100 °C to evaluate the dimensional stability [54]. The CTE is shown in the insertion thumbnail with the corresponding values of 405 × 10^−6^ K^−1^, 320 × 10^−6^ K^−1^, and 272 × 10^−6^ K^−1^, respectively. The significantly different thermal expansion behavior between these samples can be explained from the perspective of structural composition, in which the increase in crystallinity leads to an increase in rigid ECCs layers, which constrains the remaining amorphous nanophase structures to a higher stiffness at increasingly smaller size conditions [55]. Therefore, a better dimensional stability depends largely on the BONs framework.

TG and DTG thermograms of the BONs samples were obtained with the degradation being monitored as a function of temperature (Figure 5c,d). Selected temperatures of characteristic thermal parameters were the 5% weight loss, 50% weight loss, and maximum degradation temperature, which are summarized in Table 1. When the temperature was increased, sample BONs-1 first began to decompose and exhibit a two-stage decomposition. Whilst the second stage started at 397.32 °C, in which we observed the decomposition of the main body, the initial decomposition stage (<5%) at 335–345 °C can be attributed to the defects and breaks of polymer chains in the material. At the same time, the TGA curves of the rest samples indicated that the decomposition temperature was getting higher. As expected, the more layers of ECCs, the smaller was the structure of amorphous nanophase and the higher was the decomposition temperature. In addition, the maximum degradation temperature (Tmax) also tends to be consistent, that is, similar results of decomposition behavior and char yields were observed, which were in keeping with the fact of identical molecular composition of the three samples. It is gratifying to note that the formation of BONs framework exhibits superior thermal stability.

## 5. Conclusions

In summary, we proposed and prepared a multiscale framework in polymers to achieve the anisotropic enhancement of TCs by using a dual-assembly strategy. We demonstrated that the successive pressurization-stretching operation can not only rearrange the crystallite lattices into highly ordered ECCs architecture in the in-plane direction, but also further reduce the structure size of amorphous nanophases among ECCs lamellae until forming cross-planar alignments. As a result, the as-prepared BONs-3 film exhibited enhanced a TC of 4.35 $Wm^{-1}\;K^{-1}$ in the in-plane direction ($K_{∥}$) and 1.99 $Wm^{-1}\;K^{-1}$ in the through-plane direction ($K_{⊥}$). To the best of our knowledge, this is the first report on the application of anisotropic amorphous nanophase structures to enhance the TCs of pure polymers at multi-scale levels. In addition, the BONs-3 film not only exhibits the fastest one-dimensional thermal response speed in the in-plane direction, but also preforms excellent heat exchange capacity of a heat transfer coefficient up to 1241 $W/\left(m^{2}\;K\right)$. This work provides insights for the design and preparation of low-cost high TC polymer films through a relatively simple and economical method for constructing a polymer chain-based multiscale thermally conductive framework.

## Figures and Tables

**Table 1:** Thermal performance data for BONs samples.

Sample	Td5% (N_2_, °C)	Td10% (N_2_, °C)	Td50% (N_2_, °C)	Maximum Degradation Temperature (N_2_, °C)	Char Yield at 800 °C (N_2_, %)	CTE (10^−6^, K^−1^)
BONs-1	421.51	440.62	471.09	451.56	24.12	405.47
BONs-2	440.35	453.11	478.22	469.31	23.52	320
BONs-3	448.12	459.42	482.02	477.87	24.68	270

## Data Availability

The authors confirm that the data supporting the findings of this study are available within the article [and its electronic supplementary materials].

## Supplementary Materials

The following supporting information can be downloaded at: https://www.mdpi.com/article/10.3390/polym15102331/s1, Figure S1: Photo of TC testing apparatus; Figure S2: Details of plate heat exchange apparatus; Figure S3: Schematic of experimental apparatus for heat exchange measurements; Figure S4: Photo of home-made experimental setup for heat exchange testing; Table S1: Comparison of different thermal conductivity test methods [56,57,58,59,60,61,62,63,64,65,66,67,68,69,70,71,72,73,74,75,76,77,78,79].
