# Supplementary material for: Thermal Conductivity of Polyvinylidene Fluoride Films with a Multi-Scale Framework
Source: Polymers (Basel). 2023 May 16;15(10):2331. doi: 10.3390/polym15102331 (PMC10221381; doi:10.3390/polym15102331)
Supplement: Supplementary file 1 [file polymers-15-02331-s001.zip › polymers-2332032-supplementary.pdf]

Supplementary Information for

Thermal conductivity of polymer film with multi-scale framework

Qin Wang et al.

Supplementary Note 1. Overview of the methods for thermal conductivity determination

The measurement techniques for thermal conductivity can be divided into two groups: steady-state methods and transient methods. The steady-state methods determinate thermal conductivity based upon the measurement of a heat flux and a temperature gradient, and the temperature variations throughout the samples are independent of time (except for small random fluctuations). The transient methods are to measure thermal diffusivity by recording temperature as a function of time after adding transient or periodic heat to the sample surface. Transient methods are much faster than steady-state methods, but usually have lower accuracy and require more complex data analysis. Table 1 below provides an overview of the main methods for measuring thermal conductivity.

Table S1 Comparison of different thermal conductivity test methods [1]

Methods		Temperature range (K)	Conductivity range (W/mK)	Accuracy	Materials	Test standards
Steady-state methods	Guarded hot plate method[2-5]	80-800	< 0.8	2%	Glass, polymers and insulation materials	ASTM C177<br>ISO 8302<br>EN 12667
	Axial flow method[6-8]	90-1300	0.2-200	2%	Polymer, ceramics, metals	ASTM E1225
	Heat flow meter Method [5,9-11]	253-523	< 10	3%	Glass, polymers and insulation materials, ceramics	ASTM C 518<br>ASTM E1530<br>ISO 8301<br>EN 12667
	Pipe method[2,12]	293-2770	0.02–200	2%	Metals, high conductivity inorganics,	ISO 8497

					polymer composites	
	Laser flash method [2,13-16]	373–3273	> 0.01	3-5%	Glasses, polymers, ceramics, metals	ASTM E1461 ISO 22007-4 ISO 18755
Transient methods	Transient hot wire method [2,17-21]	293–2273	< 25	1-10%	Glasses, polymers, ceramics, most of liquid, gas, powders	ASTM-C1113 ISO 8894-1 ISO 8894-2
	Transient plane source method [22-25]	20–1273	0.005–1800	5%	Insulation materials, powders, polymers, ceramics, metals, liquids	ISO 22007-2

It has been demonstrated by Santos and co-workers that the laser-flash thermal analysis is suitably for evaluating the thermal properties of polymeric materials [26]. The laser-flash technique was first introduced by Parker et al. in 1961 and is commonly used to measure the thermal diffusivity through samples [27]. Therefore, we adopt laser-flash method for thermal conductivity determination in this article.

Thermal conductivity of all samples is measured by a laser flash method using NETZSCH LFA 467 NanoFlash instrument (NETZSCH Leading Thermal Analysis, No:15010459, Germany). For measuring through-plane thermal conductivity, film samples are cut into square plates with the size of 10 mm×10 mm and each sample is tested three times. The testing samples are coated by a graphite layer of around 5 μm on both upper and lower surfaces, ensuring that all parts of the sample have equal light energy absorption ratio and infrared emissivity. The test is operated in through-plane mode and the curve is fit with the "Cowan & pulse correction" model.

The thermal diffusivity is determined by analyzing the temperature versus-time curve based on the following equation:

$$\alpha = \frac{0.1338d^2}{t_{50}}$$

where α is the thermal diffusivity, d is the thickness of the tested sample,and t50 is half of the diffusion time. Accordingly, the thermal conductivity (κ) could be calculated using following equation:

$$\kappa = \alpha \times C_p \times \rho$$

where Cp is the specific heat capacity, measured using differential scanning calorimetry (Q200,TA Instrument). ρ is the density of samples,which is obtained according to ρ=m/V, among which, m and V are the mass and volume of the sample, respectively. The Cp and ρ are obtained by taking the average value of five separate measurements.

The in-plane thermal conductivity is measured by using Laminar method. Firstly, samples are cut into strips with the size of 10 mm ×2 mm. Then, rotating each strip by 90° (the original horizontal direction becomes the vertical direction), and placing the strips tightly to each other in the laminar bracket. Finally, the testing samples are carefully coated with a graphite layer on both upper and lower surfaces. The test and data analysis process are the same as the common through-plane test.

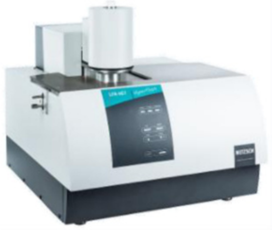

Figure S1 Photo of TC testing apparatus.

Supplementary Note 2. Experimental of heat exchanger applications

S2.1 Experimental apparatus of homemade plate heat exchanger

The experiment of heat exchanger application was carried out with a homemade heat exchanger which assembled by two polytetra-fluoroethylene frameworks sandwiched with polyvinylidene fluoride films (FIG. S2a-c).

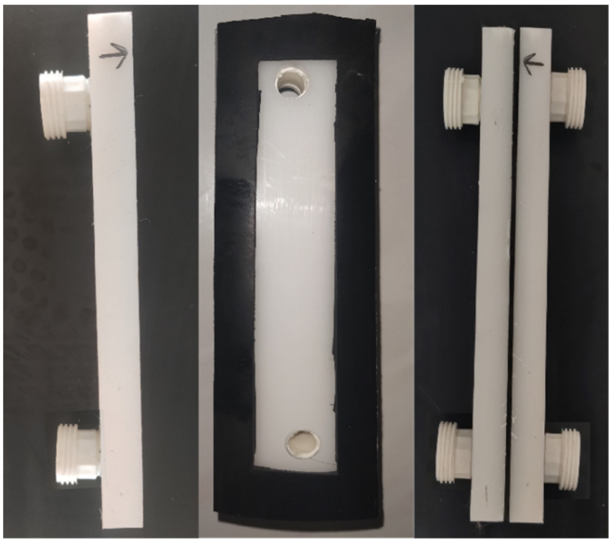

Figure S2 Details of plate heat exchange apparatus. (a) Side view of plate heat exchanger framework, (b) top view of plate heat exchanger framework with rubber seal, (c) side view of plate heat exchanger apparatus.

S2.2 Experimental apparatus and procedure

As shown in Figure S3, the apparatus is composed of a homemade heat exchanger and two independent cold and hot water loops. Each loop is driven by a diaphragm pump and maintained at a constant temperature through a thermostatic bath. The fluid temperature was measured by thermocouples (PT 100) with an accuracy of ± 0.1 °C, and the flow rates of the liquid system were carried out using

flow meters with a relative uncertainty of ± 1.5%. The experimental data are obtained after 2 hour steady-state operation. The flow rates of the hot water and cool water were set to 70 L/h and 140 L/h, respectively. The temperature of hot water and cool water were set to 70 °C and 15 °C, respectively. The Photo of home-made experimental setup is shown in Figure S4.

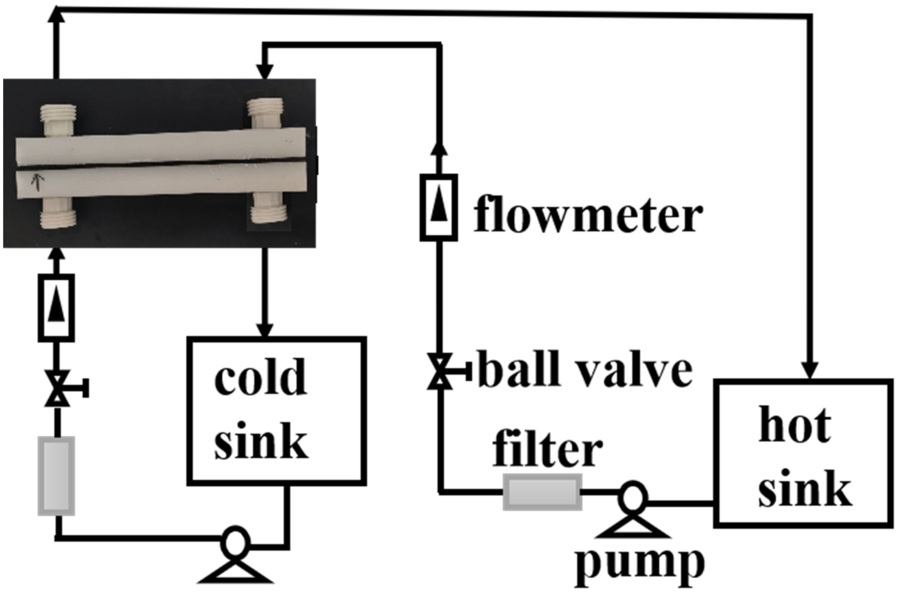

Figure S3 Schematic of experimental apparatus for heat exchange measurements.

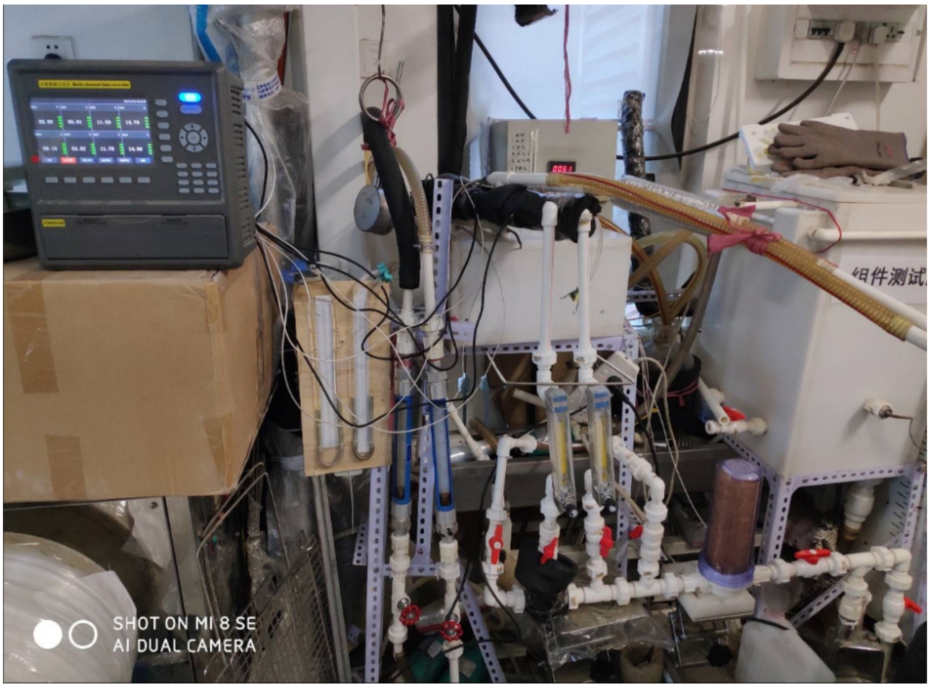

Figure S4 Photo of home-made experimental setup for heat exchange testing.

Supplementary Note 2. Equations and Nomenclature

S2.1 Equations for the calculation of overall heat transfer coefficients

The following equations are used to calculate the overall heat transfer coefficient (U).

$$Q=mc_p\Delta T=UA\Delta T_{lm} \quad (1)$$

$$\Delta T_{lm}=\frac{(T_{h,in}-T_{c,out})-(T_{h,out}-T_{c,in})}{\ln\frac{T_{h,in}-T_{c,out}}{T_{h,out}-T_{c,in}}} \quad (2)$$

$$A=ab \quad (3)$$

S2.2 Experimental apparatus of homemade plate heat exchanger

Nomenclature

Symbols

A	heat transfer area, m^2
a	Effective length of heat exchange surface, m
b	Effective width of heat exchange surface, m
c_p	specific heat of fluid, J/ (kg K)
L	the effective length of the hollow fibers, m
m	mass flow rate of loop water, kg/S
Q	rate of heat transfer, W
$\triangle T$	temperature difference of loop water, °C
$\triangle T_{lm}$	logarithmic mean temperature, °C
$T_{c,in}$	cold water inlet temperature, °C
$T_{c,out}$	cold water outlet temperature, °C
$T_{h,in}$	hot water inlet temperature, °C
$T_{h,out}$	hot water outlet temperature, °C
U	overall heat transfer coefficient, W/ (m^2K)

[1] *Chen H. et al. Progress in Polymer Science 59 (2016) 41–85.*
[2] *Buck W, Rudtsch S. Thermal properties. In: Czichos H, Saito T, Smith L, editors. Springer handbook of materials measurement methods. Berlin, Heidelberg: Springer; 2006. p. 399–429.*
[3] *Anonymous. Standard test method for steady state heat flux measurements and thermal transmission properties by mean of the guarded hot plate apparatus. ASTM C177-13: American Society for Testing and Materials; 2013, 23 pp.*
[4] *Anonymous. Determination of steady-state thermal resistance and related*

properties – guarded hot plate apparatus. International Standard ISO 8302:1991; 2013, 47 pp.
[5] *Anonymous. Thermal performance of building materials and products – determination ofthermal resistance by means of guarded hot plate and heat flflow meter methods – products of high and medium thermal resistance. EN 12667: 2001. European Standard; 2001, 52 pp.*
[6] *Corsan J M. Axial heat flflow methods of thermal conductivity measurementfor good conducting materials. In: Maglic KD, Cezairliyan A, Peletsky VE, editors. Compendium of thermophysical property measurement methods. Recommended measurement techniques and practices, vol. 2. New York: Plenum Press; 1992. p. 3–31.*
[7] *Maglic K D, Cezairliyan A, Peletsky V E. Compendium of thermophysical property measurement methods, vol. 1: Survey of measurement techniques. New York: Plenum Press; 1984. p. 11–40.*
[8] *Anonymous. Standard test method for thermal conductivity of solids using the guarded-comparative-longitudinal heat flflow technique,ASTM E1225-13.American Society for Testing and Materials; 2013, 10 pp.*
[9] *Anonymous. Standard test method for steady-state thermal transmission properties by means of the heat flflow meter apparatus, ASTM C518-10. American Society for Testing and Materials; 2010, 16 pp.*
[10] *Anonymous. Standard test method for evaluating the resistance of thermal transmission of materials by the guarded heat flflow meter technique,ASTME1530-11.American Society for Testing and Materials; 2011, 9 pp.*
[11] *Anonymous. Thermal insulation – determination of steady-state thermal resistance and related properties – heat flflow meter apparatus. International Standard ISO 8301:1991; 2014, 38 pp.*
[12] *Anonymous. Thermal insulation – determination of steady-state thermal transmission properties of thermal insulation for circular pipes. International Standard ISO 8497:1994; 2013, 16 pp.*
[13] *Anonymous. Standard test method for thermal diffusivity by the flash method, ASTM E1461-13. American Society for Testing and Materials; 2013, 11 pp.*
[14] *Anonymous. Plastics – determination of thermal conductivity and thermal diffusivity – Part 4; Laser flflash method. International Standard ISO 22007-4:2008; 2011, 12 pp.*
[15] *Anonymous. Fine ceramics (advanced ceramics, advanced technical ceramics) – determination of thermal diffusivity of monolithic ceramics by laser flflash method. International Standard ISO 18755:2005; 2014, 31 pp.*
[16] *Anonymous. An overview of the various thermal conductivities depending on the used method. Netzsch Company; 2015.*
[17] *Anonymous. Standard test method for thermal conductivity of refractories by hot wire (platinum resistance thermometer technique), ASTM C1113/C1113M – 09(2013). American Society for Testing and Materials; 2013, 6 pp.*
[18] *Anonymous. Refractory materials – determination of thermal conductivity – Part 1; Hot-wire methods (cross-array and resistance thermometer). International Standard ISO 8894-1:2010; 2015, 19 pp.*
[19] *Anonymous. Refractory materials – determination for thermal conductivity – Part 2; Hot-wire method (parallel). International Standard ISO 8894-2:2007; 2011, 13 pp.*
[20] *Vozar L. A computer-controlled apparatus for thermal conductivity measurement*

by the transient hot wire method. J Therm Anal 1996;46:495–505.
[21] Kwon SY, Lee S. Precise measurement of thermal conductivity of liquid over a wide temperature range using a transient hot-wire technique by uncertainty analysis. Thermochim Acta 2012;542:18–23.
[22] Solorzano E, Rodriguez-Perez MA, de Saja JA. Thermal conductivity of cellular metals measured by the transient plane source method. Adv Eng Mater 2008;10:596–602.
[23] Gustafsson SE. Transient plane source techniques for thermalconductivity and thermal-diffusivity measurements of solid materials. Rev Sci Instrum 1991;62:797–804.
[24] Anonymous. Instruments comparison chart (hot disk AB); 2015.
[25] Anonymous. Plastics – determination of thermal conductivity and thermal diffusivity – Part 2; Transient plane heat source (hot disk) method. International Standard, ISO 22007-2:2015; 2015, 20 pp.

[26] Dos Santos W N, Mummery P, Wallwork A. Thermal diffusivity of polymers by the laser flflash technique, Polym. Test. 24 (2005) 628-634.
[27] Parker W J, Jenkins R J, Butler C P, Abbott G L. Flash method of determining thermal diffusivity, heat capacity and thermal conductivity. J. Appl. Phys. 32 (1961) 1679-1684.
